# Gene suppression approaches to neurodegeneration

**DOI:** 10.1186/s13195-017-0307-1

**Published:** 2017-10-05

**Authors:** Rhia Ghosh, Sarah J. Tabrizi

**Affiliations:** UCL Huntington’s Disease Centre, Department of Neurodegenerative Disease, UCL Institute of Neurology, London, WC1N 3BG UK

**Keywords:** Gene suppression, RNA interference, Anti-sense oligonucleotides, Zinc-finger proteins, CRISPR/Cas9, Therapeutics, Huntington’s disease, Dementia

## Abstract

Gene suppression approaches have emerged over the last 20 years as a novel therapeutic approach for the treatment of neurodegenerative diseases. These include RNA interference and anti-sense oligonucleotides, both of which act at the post-transcriptional level, and genome-editing techniques, which aim to repair the responsible mutant gene. All serve to inhibit the expression of disease-causing proteins, leading to the potential prevention or even reversal of the disease phenotype. In this review we summarise the main developments in gene suppression strategies, using examples from Huntington’s disease and other inherited causes of neurodegeneration, and explore how these might illuminate a path to tackle other proteinopathy-associated dementias in the future.

## Background

Gene suppression approaches refer to targeted molecular genetic therapies that serve to lower the expression of specific genes. The term “gene silencing” has also been used to describe these methods; however, this term should be considered a misnomer as complete gene inactivation does not occur, and might not be desirable. Such techniques have made enormous progress over the last 20 years, and show great promise for the treatment of inherited neurodegenerative diseases arising from a known genetic mutation.

Neurodegenerative diseases that result from the toxic gain-of-function of a mutant protein or non-coding RNA that are without a significant loss-of-function are ideal candidates for gene suppression approaches. There are many strategies to lower the amount of toxic disease proteins that result from single gene mutations, including both post-transcriptional inhibition such as RNA interference (RNAi), anti-sense oligonucleotides (ASOs) and catalytic nucleic acids, and genome editing techniques such as zinc finger proteins (ZFPs) and CRISPR/Cas9. The great benefit of interventions at the gene suppression level, as opposed to interventions aimed at the toxic protein itself, is that the plethora of potentially negative downstream cellular pathogenic effects that may arise from the abnormal functioning of a single protein are all reduced as a consequence of treatment.

In this review we will use examples from Huntington’s disease (HD), in respect of which a worldwide collaborative research effort has led to the swift progression of gene suppression approaches, from in-vitro and in-vivo development through to a clinical trial of a Huntingtin-lowering therapy that is currently in progress [1]. Huntington’s disease is an autosomal dominant, neurodegenerative condition caused by a CAG expansion in exon 1 of the gene encoding the Huntingtin (HTT) protein. The presence of the mutant gene leads to the adult onset of chorea, psychiatric symptoms and cognitive decline, and is fatal after 15–20 years [2]. In addition, examples of gene suppression approaches in amyotrophic lateral sclerosis (ALS) [3] will be discussed. However, lessons learnt from development of gene suppression technologies in these conditions could equally be applied to any dementia or neurodegenerative condition in which the responsible disease protein is known. Indeed the potential of lowering tau protein for the treatment of Alzheimer’s disease (AD) and other tauopathies has been demonstrated recently [4], as well as targeting alpha-synuclein (SNCA) [5] and leucine-rich-repeat kinase 2 (LRRK2) [6] for Parkinson’s disease (PD), and ataxin-2 for the treatment of spinocerebellar ataxia type 2 (SCA2) [7], sporadic ALS and frontotemporal dementia (FTD) [8].

## Post-transcriptional gene suppression

**Fig. 1:**
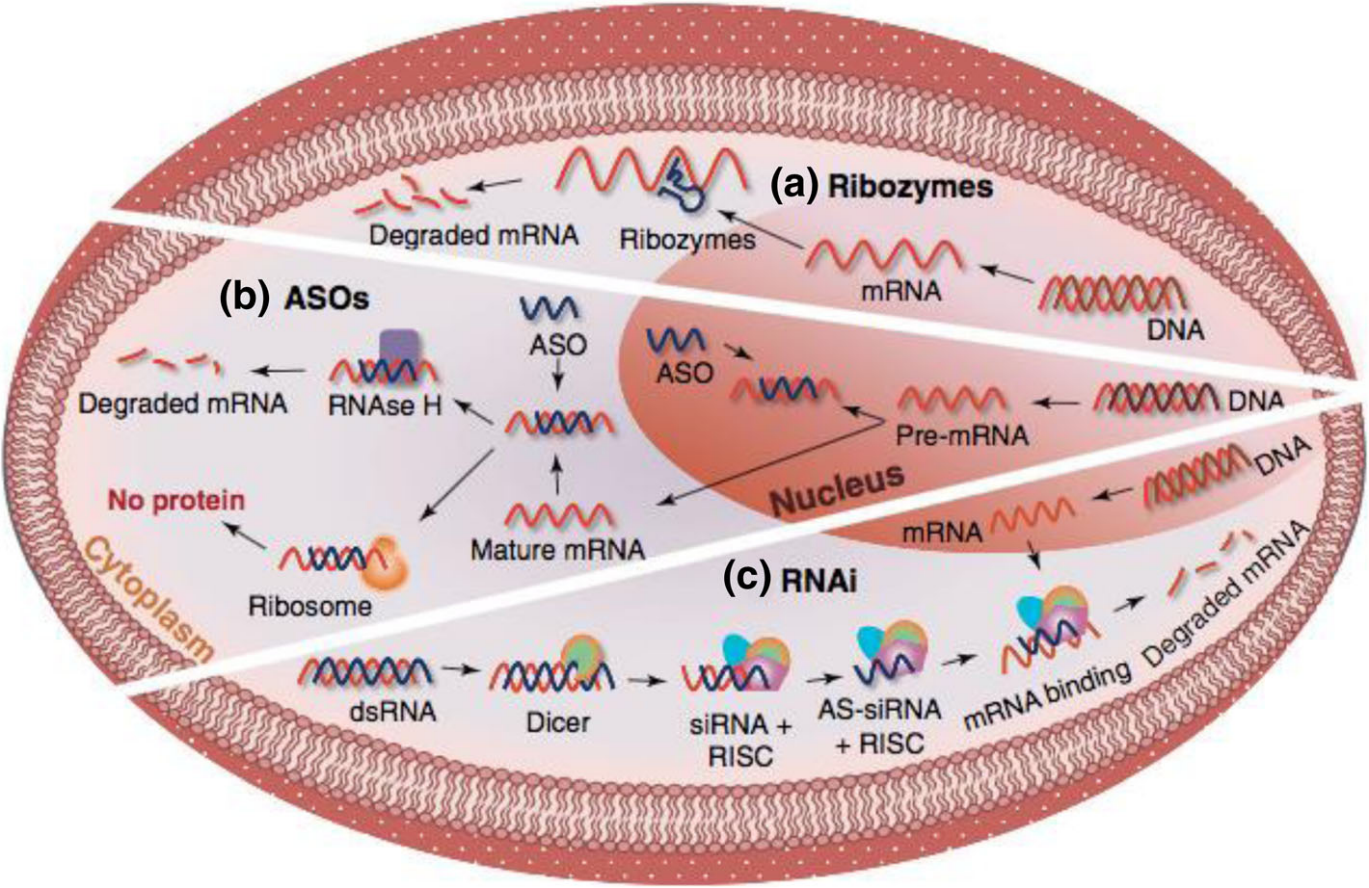
Mechanisms of post-transcriptional gene suppression. **a** Ribozymes act in the cytoplasm where they hybridise complementary mature mRNA sequences and induce catalytic cleavage. **b** ASOs bind to complementary mRNA targets, leading to RNAse H1-induced mRNA cleavage. They are able to target both pre-mRNA in the nucleus and mature mRNA in the cytoplasm. **c** RNAi occurs in the cytoplasm and leads to the degradation of mature mRNA via a complex and highly regulated process. ASO: anti-sense oligonucleotide, AS-siRNA: antisense short interfering RNA, RISC: RNA-induced silencing complex, RNAi: RNA interference (Reproduced from Godinho et al. [9] with permission from Elsevier)

Post-transcriptional gene suppression refers to approaches that trigger the cleavage, enhanced degradation or translational suppression of the target mRNA. They include RNAi, ASOs and catalytic nucleic acids (ribozymes and DNA enzymes). Ultimately all of these mechanisms serve to modulate translation efficiency, thus lowering the amount of protein expressed [9] (see Fig. 1).

### RNA interference

**Fig. 2:**
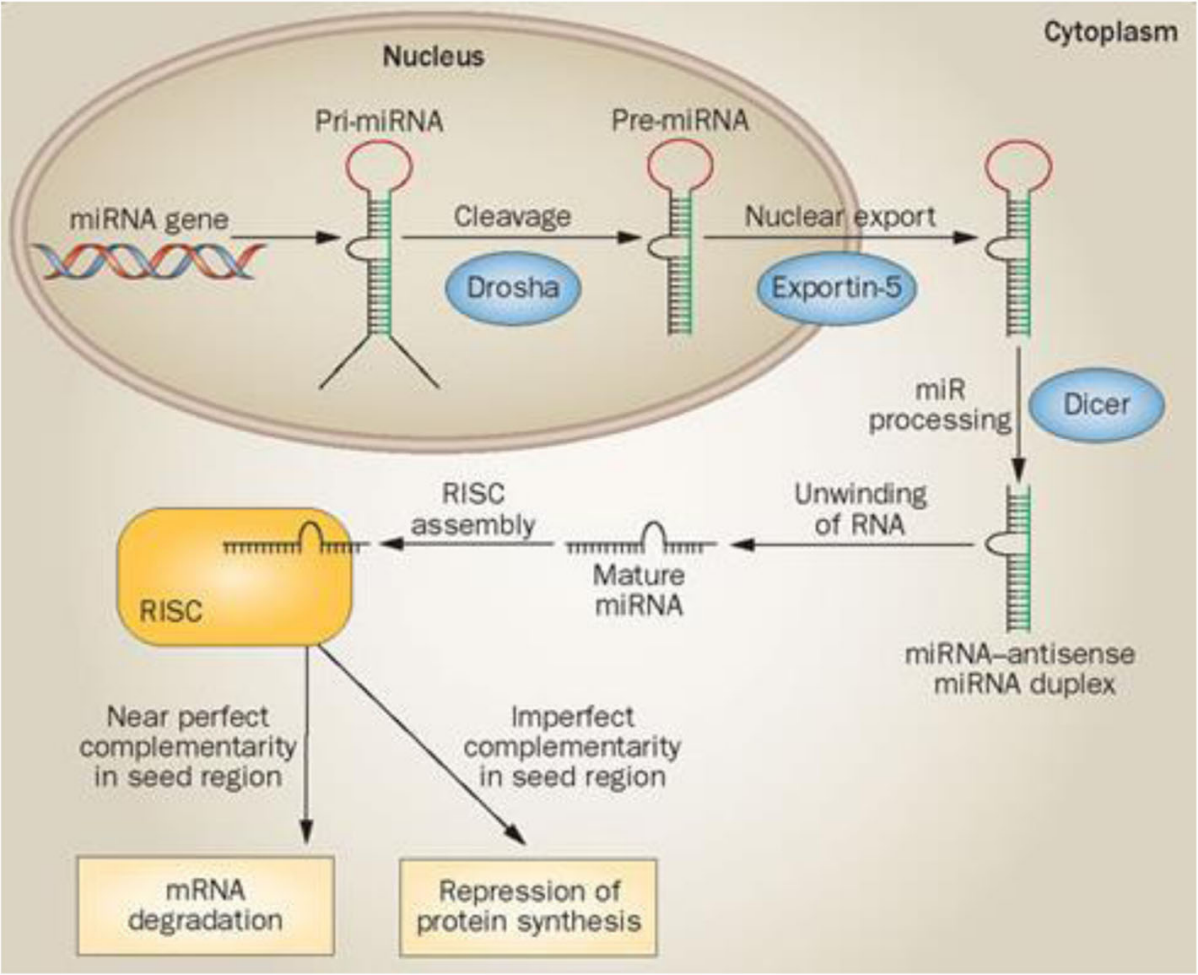
miRNA processing pathway. miRNA: microRNA, pre-miRNA: precursor miRNA, pri-miRNA: primary miRNA, RISC: RNA-induced silencing complex (Reproduced from O’Kelly et al. [101] with permission from Nature Publishing Group)

Endogenous RNAi is an evolutionarily conserved process within cells, whereby native, non-coding, double-stranded RNA sequences elicit post-transcriptional gene suppression, typically by causing the destruction of specific mRNA molecules, thereby regulating mRNA expression [10] (see Fig. 2). Two types of small RNA molecules—endogenous genomically non-coding RNAs called microRNA (miRNA) and exogenous short-interfering RNA (siRNA)—can bind to other specific mRNA molecules and increase or decrease their activity.

This well-described mechanism is comprised of a number of steps [11]:

1. Within the nucleus, miRNAs are transcribed by RNA polymerase II (pol II) and pol III promoters to form stem–loop structures known as primary miRNAs (pri-miRNAs).
2. pri-miRNAs are cleaved by the endoribonuclease enzyme Drosha to form a precursor miRNA (pre-miRNA) hairpin-like structure.
3. pre-miRNAs are exported to the cytoplasm by Exportin-5.
4. In the cytoplasm, the pre-miRNA is further processed by another endoribonuclease enzyme Dicer to create a mature miRNA complex.
5. The antisense or “guide strand” of the (endogenous miRNA or exogenous siRNA) complex is loaded into the RNA-induced silencing complex (RISC), which is located in the cytoplasm, and the sense or “passenger” strand is degraded.
6. The guide strand targets the RISC to its complementary mRNA. Perfect base pair matching in the case of siRNA leads to Argonaut-2 (Ago-2)-mediated cleavage of the target and complete translational inhibition. Imperfect complementarity in the case of miRNA leads to translational repression.

siRNAs differ from miRNAs in that miRNAs typically have incomplete base pairing to a target and inhibit the translation of many different mRNAs with similar sequences. siRNAs, in contrast, typically base-pair perfectly and induce mRNA cleavage only in a single, specific target.

Intensive research has been carried out to generate effectors that manipulate the RNAi pathway to suppress the expression of a particular gene of interest. These have taken the form of siRNAs that include short hairpin RNAs (shRNAs), and artificial miRNAs, all of which aim to degrade mature, spliced mRNA in the cytosol.

siRNAs are comprised of 20–25 base pairs, are processed by Dicer and ultimately lead to Ago-2-mediated cleavage of the target mRNA, to interfere with the expression of specific genes with complementary nucleotide sequences. They can be delivered into cells in liposome formulations [12], nanoparticles [13] or as chemically modified single-stranded siRNAs [14, 15]. siRNAs do not cross an intact blood–brain barrier (BBB), are not soluble in artificial CSF and when injected directly into the brain do not typically penetrate the plasma membrane without modification.

Researchers have therefore used RNA expressed from viral gene therapy vectors to achieve stable expression in brain regions of interest. The RNA from the viral genome is processed by Dicer to RNAi (e.g. shRNAs and artificial miRNAs). shRNAs delivered into cells using viral vectors have been shown to cause extremely high levels of translational suppression [16, 17], although this has also caused saturation of the endogenous RNAi machinery [18]. More recently, siRNAs have been embedded into artificial miRNA backbones to achieve mRNA suppression without the build-up of antisense precursors in the cell [11].

#### RNAi as therapeutics in neurodegenerative disease

RNAi has been investigated widely in HD, and many potential scientific hurdles have been overcome. It is worth bearing in mind that the underlying principles of RNAi for the potential treatment of HD could also be applied to other causes of dementia. Initially, RNAi to lower the amount of Huntingtin protein (HTT) was shown in vitro to improve cell survival in cellular models of HD [19]. In 2005 the first in-vivo studies of RNAi were carried out. Single bilateral injections of AAV-encased anti-HTT shRNA were delivered into the striatum of HD transgenic mice (N171-82Q), leading to significant reductions in both mutant *HTT* mRNA levels and in the formation of HTT inclusions (which are a pathognomic feature of HD) [20]. Improvements in certain motor symptoms were also found. Similar results have also been reported in other mouse models of HD (R6/1), with increased presence of striatal markers (DARPP-32) and preproenkephalin (ppENK) following treatment [16].

Non-viral delivery systems have also been tested, using nanoparticles to deliver lipid formulated siRNAs via the intracerebral ventricular route in the R6/2 mouse model of HD. This lowered mutant *HTT* mRNA levels and inclusions, and reduced brain atrophy. Sustained effects including delayed onset of motor symptoms and improved survival were seen [21]. Delivery of cholesterol-conjugated siRNA duplexes targeting human HTT mRNA into the striatum have also shown feasibility and efficacy in an AAV mouse model of HD [22].

These studies all administered treatment in pre-symptomatic HD mice, but treatment in symptomatic HD mice has also successfully reduced HTT protein levels, reduced inclusions and improved histopathology [23]. The impact on HD behavioural deficits and any potential reversal of phenotype remain to be seen. In the case of prion disease, hippocampal delivery of lentivirus expressing shRNA targeting prion protein (PrP) significantly prolonged survival of mice with established prion disease. The onset of behavioural deficits was prevented and spongiform degeneration decreased when the same treatment was administered to mice with early prion disease [24]. This highlights the importance of the timing of gene suppression therapies in the treatment of neurodegenerative disorders.

The earlier siRNA studies in HD animal models did not affect the endogenous levels of wild-type HTT protein—only the disease-causing transgene was reduced. In addition, the models were also all expressing a small N-terminal fragment of the mutant protein (thought to be the potential disease-causing species in HD [25]) and not full-length mutant HTT. Attempting to target the full-length mutant *HTT* mRNA, whilst leaving the mRNA of the wild-type allele unaffected, has proven to be very difficult in cells derived from HD patients (although newer strategies are being developed to try and do just that—see later). An alternative approach is to non-specifically lower both mutant and wild-type HTT to a degree which improves pathology whilst maintaining safety and tolerability in the face of wild-type HTT loss. A 40–60% reduction in both endogenous and mutant HTT extends the lifespan and prevents motor deficits, without causing toxicity in both fragment [26] and full-length [17] mouse models of HD. Again, this was also found to be the case when treating symptomatic HD rodents [27, 28].

RNAi testing in HD non-human primates (adult rhesus monkeys) has also demonstrated the safety of partially reducing endogenous wild-type HTT following delivery of AAV vectors expressing artificial miRNAs and shRNAs. A 45% reduction in wild-type HTT did not induce any pathology or symptomatology [29] and sustained knock-down was still evidenced and well tolerated after 6 months [30]. These findings have since been replicated in further studies showing that direct infusion of siRNA into the rhesus putamen lowered HTT to a similar degree and that there was a partially sustained effect up to 39 days post treatment [31, 32]. Therefore partial lowering of both mutant and wild-type HTT may be a viable treatment strategy in HD.

Translation of this finding to other genetic forms of neurodegenerative disease is not assured. The consequences of lowering levels of endogenous wild-type protein needs to be investigated carefully when non-allele-specific targeting is considered for conditions in which only the mutant protein is pathogenic. However, for neurogenetic disorders in which misfolding of the mutant protein corrupts native/wild-type protein, recruiting them to spread pathology, non-allele-specific targeting may be required for pharmacologic effect.

### Anti-sense oligonucleotides

ASOs are single-stranded DNA molecules consisting of 16–22 bases, which are complementary to the target pre-mRNA. ASOs have a more upstream site of action than RNAi effectors that act on mature, spliced mRNA species. Upon binding, ASOs recruit RNaseH1 (an endogenous enzyme that recognises RNA/DNA duplexes), which degrades the target pre-mRNA. Alternatively, ASOs may obstruct the interactors of the pre-mRNA leading to translational inhibition or modulation of splicing [11].

Over the last 20 years, modifications of ASOs have improved their suitability for therapeutic application in neurodegenerative diseases. These modifications include the following:

1. Sulphur substitution for oxygen in the phosphate of the phosphate inter-nucleotide linkages or backbone of the ASO molecule, thus increasing resistance to nucleases and improving protein binding.
2. Multiple potential alterations to the sugar moiety of the nucleotide which increase binding affinity to the target mRNA, increase resistance to nucleases and decrease toxicity to varying degrees. Importantly, most sugar modifications lead to failure to recruit the RNaseH1 enzyme. Therefore, when RNaseH1 activity is desired, nucleotides at the ends of the ASO can be modified to improve binding affinity and resistance to nucleases, while leaving a stretch of unmodified nucleotides in the middle that can engage RNaseH1.

Engineering ASOs with specific desired characteristics can be achieved by incorporating one or more of these chemical modifications into their design. A diverse range of ASOs is therefore possible, with different potencies, pharmacokinetics and pharmacodynamics, as well as different targets.

#### ASOs as therapeutics in neurodegenerative disease

ASOs are currently being tested in early-stage clinical trials in patients with HD and ALS [3], and have been approved by the US Food and Drug Administration (FDA) for the treatment of patients with spinal muscular atrophy (SMA) [33, 34] (see section “Recent and ongoing clinical trials of gene suppression”). In the case of HD, since September 2015 an ASO with a modified sugar group (MOE) targeting human HTT mRNA has been delivered by lumbar intrathecal bolus injection to patients with early-stage HD as part of a phase 1/2a clinical trial sponsored by Ionis Pharmaceuticals [1].

This ASO has been tested in pre-clinical studies and is predicted to lower total HTT levels in a dose-dependent manner. It has been shown to reverse behavioural phenotypes in BACHD and YAC128 mouse models of HD [35] and to ameliorate key striatal gene expression changes (YAC128) [36]. As was the case with RNAi, non-allele-specific suppression with subsequent lowering of endogenous HTT did not cause any toxicity. Phenotypic improvements were greatest when treatment was initiated earlier in the disease course, and were sustained for many months following termination of treatment [37]. Suppression of the target mRNA itself also lasted for 12 weeks after a single administration of ASO in these mice [35]. This has enormous implications for the timing of intervention and the dosing frequency that would be required for HD patients; this is of particular importance given the relatively invasive route required for drug administration (see later).

### Catalytic nucleic acids

#### Ribozymes

Ribozymes are naturally occurring RNA molecules comprising two flanking sequences that enable specific binding to target mRNA, and an effector catalytic core that cleaves the mRNA substrate. The mRNA fragments are then degraded by the cell [9].

Hammerhead ribozymes (a specific class of ribozymes 30–40 nucleotides in length), delivered using an AAV vector, have been shown to result in 60% reduction of mHTT mRNA when delivered to HEK293 cells expressing mHTT exon 1 [38]. Direct striatal injection of these ribozymes into a transgenic HD mouse (R6/1) also led to a 30% reduction of mHTT RNA in the mouse brain [38], but the impact on motor and behavioural symptoms has not been studied. AAV delivery of a ribozyme against alpha-synuclein into the substantia nigra of a rat model of PD reduced alpha-synuclein protein levels and neuronal loss [39]. Finally, a new generation of ribozymes (hepatitis delta virus (HDV)) has been used in neuroblastoma cells to reduce the expression of amyloid precursor protein (APP) by 70% and the total secretion of amyloid-beta peptides by 30%, thus raising the possibility of ribozymes as therapeutics in AD [40].

#### DNAzymes

DNAzymes are synthetic single-stranded catalytic nucleic acids that also have substrate binding arms to target complementary mRNA and a cation-dependent catalytic core. They have been shown to knock-down mutant *HTT* mRNA constructs by 85% in a HEK293 cell model of HD [41], but further research in this area is needed.

## Genome editing for gene suppression

Gene suppression can also be achieved at the transcriptional level, using effectors that bind to specific DNA sequences. One such approach uses ZFPs which contain a zinc finger domain that can be manipulated synthetically to bind a DNA sequence of interest, fused to a functional protein domain. Zinc finger nucleases (ZFNs) cleave DNA at a particular target site, and zinc finger transcriptional repressors (ZFTRs) are transcription factor DNA-recognition motifs fused to a transcriptional repressor domain. Delivery of such agents would be through the use of viral vectors.

Potential advantages of this approach are the avoidance of on-target and off-target toxicities of RNA therapeutics and the elimination of all disease-causing mRNA splice variants, not just those that possess the target mRNA sequence [42]. The prospect of germ-line treatment, which would benefit future generations, is also possible. In the case of HD, ZFPs delivered intra-parenchymally using AAV vectors in a transgenic mouse model of HD (R6/2) reduced mutant *HTT* expression (without affecting wild-type *HTT* expression) and led to improvements in neuropathology and motor deficits [43]. The pharmaceutical company Sangamo, in conjunction with Shire Pharmaceuticals, are planning to take this approach forwards into clinical trials.

Other engineered nucleases include mega-nucleases, transcription activator-like effector nucleases (TALENs) and clustered regulatory interspaced short palindromic repeat (CRISPR)/Cas9 systems [44]. CRISPR/Cas9 is an endogenous mechanism within prokaryotic cells that recognises and destroys foreign DNA. This system can be manipulated to incorporate a synthetic guide RNA (gRNA) strand that targets a particular DNA location for cutting, followed by the insertion of DNA (e.g. stop codons) that essentially inactivates the mutant allele [45]. The technique has been demonstrated successfully in HD patient-derived fibroblasts, leading to the dramatic reduction of mutant HTT RNA and mHTT protein [46].

In addition to repressing gene transcription, the ability of these methods to target nuclease-induced scission and repair offers the exciting possibility of actually correcting genetic mutations (e.g. through the excision of the expanded CAG repeat in HD)—leading to true genome editing. There is thus the exciting potential to treat not just HD but any genetic cause of neurodegenerative dementia using these techniques.

## Potential challenges in gene suppression approaches

### Drug delivery and administration

#### RNA interference

Because unmodified siRNAs do not readily cross the blood–brain barrier (BBB), or the plasma membrane of cells, researchers have used viral vectors to enable stable expression of shRNAs and artificial miRNAs within desirable brain regions. The most commonly used vectors are recombinant adeno-associated virus (AAV) and lentivirus (LV), both of which are non-pathogenic, trigger minimal immune response and cannot replicate in the host. AAVs remain as nuclear episomes and do not integrate into the host genome, but result in stable gene expression that is stronger than that of LVs. siRNA sequences constitutively delivered through miRNA expression systems expressed from a viral vector should minimise the requirement for repetitive administration. There are multiple capsid serotypes of AAV, allowing for specific cell-directed therapy [47, 48]. Unfortunately many humans have pre-existing antibodies to certain AAV serotypes that trigger a neutralising response against the virus [49, 50]. It may be possible to screen potential patients for this, and work is also being done to engineer AAV capsids that evade these antibodies.

In the case of HD the primary site of pathology is the striatum, thus RNAi treatments in animal models have generally been delivered by bilateral direct injection or infusion into the striatum, or the adjacent lateral ventricle (resulting in widespread delivery throughout the brain) [11]. However, HD also results in global brain pathology and dysfunction as well as a peripheral metabolic phenotype. Recently, RNAi treatment targeted to the hypothalamus has been shown to reduce metabolic symptoms in HD mouse models [51]. These administration techniques would require stereotactic neurosurgery in patients, which is extremely invasive and not without risk.

AAV serotype 9 (AAV9) crosses the BBB and transduces neurons and glia in the brain, as well as a variety of peripheral tissues, following a systemic, intravascular injection [52]. A global targeting approach using jugular vein injection of AAV9 expressing artificial miRNA into transgenic HD mice showed reduced expression of mHTT in the cortex, striatum, hypothalamus and hippocampus. This led to a reduction of cortical and striatal atrophy, as well as reduced inclusion formation [53]. More recently, Deverman et al. described a capsid selection method, called Cre-recombination-based AAV targeted evolution (CREATE), to generate AAV variants with high CNS tropism. Following intravenous injection in mice, the variant AAV-PHP.B transduced the majority of neurons and astrocytes across multiple CNS regions, with 40-fold greater efficacy than the current standard (AAV9) [54]. These studies raise the possibility of future peripheral administration of RNAi therapy in the treatment of brain disorders.

#### Anti-sense oligonucleotides

ASOs also do not cross the BBB, but can be delivered into the cerebrospinal fluid (CSF), either through intrathecal or intra-ventricular injection, as they are soluble in artificial CSF. Once injected, ASOs are distributed to the brain parenchyma and taken up into both neuronal and glial cells. Unlike RNAi, ASOs are not regenerated within the cell and have a half-life that varies depending on the precise sequence and chemical structure. Repeated dosing of the drugs is therefore required to sustain treatment effect. ASOs display dose dependency and reversibility, and therefore the degree of protein lowering can be controlled to achieve safe yet effective levels.

In the case of HD, ASOs delivered intra-ventricularly to rodents led to > 75% reduction of target *HTT* mRNA throughout the brain and spinal cord. In NHP brains, ASOs delivered intrathecally were found in the spinal cord, in the cortex and to a lesser extent in the deeper brain structures [35]. Although ASOs achieve widespread distribution throughout the brain after lumbar intrathecal bolus delivery into the CSF [55], the highest tissue levels are in areas adjacent to the CSF. This suggests that passive diffusion may play an important role in drug distribution, although the cardiac and respiration-associated oscillations of CSF that propel the fluid along paravascular routes may account for distribution to deeper structures. However, even in areas with low ASO levels, populations of neuronal cell bodies containing higher levels of ASO are found, suggesting that active transport (anterograde or retrograde) also plays a part. Ultimately, injection of ASOs into the CSF does lead to drug distribution across wide areas of the CNS [11].

In the future, medical devices (e.g. implantable pumps) may be able to replace the need for repeated lumbar punctures to achieve regular intrathecal dosing of ASOs. This would reduce the burden of administration for both patients and medical professionals. Recently, the development of peptide-conjugated ASOs delivered through intravenous injection (IVI) has been shown to have broad peripheral and CNS distribution in a mouse model of severe SMA. A profound improvement in mean survival from 12 to 456 days was also seen [56]. IVI is a far less invasive method of drug administration than intrathecal injection, and there is hope that this could one day prove to be an effective method of drug delivery in patients.

### Off-target effects

Off-target effects are unintended consequences following the administration of gene suppression therapies. They include interference with non-target mRNA transcripts and activation of the immune system in response to drug delivery. In the case of siRNAs and ASOs, there is potential to bind mRNA sequences that share sequence homology to the desired target, thus causing down-regulation of unrelated proteins [57]. Because structurally related microRNAs modulate gene expression largely via incomplete complementarity base pair interactions with a target mRNA, the introduction of an siRNA may cause unintended off-targeting. For ASOs, selecting unique target sequences that do not appear anywhere else in the genome can overcome this risk. In the treatment of HD, both ASOs and RNAi targeting the mRNA CAG expansion carry the possibility of affecting other PolyQ containing proteins.

When a mammalian cell encounters a double-stranded RNA such as an siRNA, the cell may mistake it as a viral by-product and mount an immune response. siRNAs have been shown to up-regulate the expression of interferon (IFN)-stimulated genes through the activation of protein kinase R (PKR) [58]. siRNAs have also been shown to activate certain toll-like receptors (TLRs), leading to the expression of pro-inflammatory cytokines [59–61]. Careful design of gene suppression effectors to generate high complementarity, and chemical modifications that enhance their stability and reduce immunogenicity, can mitigate some of these effects [9].

### Saturation of the RNAi pathway

Delivery of shRNA effectors has been shown to overload exportin-5, thus preventing the maturation of endogenous miRNA and effectively shutting down the cellular miRNA pathway [62, 63]. Saturation of the endogenous RNAi pathway can in turn lead to detrimental effects such as liver toxicity [64, 65]. This effect can be mitigated by selecting promoters for only modest expression of shRNAs, or by the co-expression of recombinant exportin-5 [64, 66]. Artificial miRNAs are better tolerated and synthetic siRNAs are able to bypass the nuclear processing step altogether [17, 67]. This particular issue does not arise when using ASOs, as the RNAi pathway is not co-opted, and ASOs have clear dose-dependent and reversible effects.

### Alternative pathways to neurotoxicity

In theory, certain gene suppression strategies may not be as effective in cases where there is evidence of abnormal RNA processing or toxicity of the RNA itself. In the case of HD, aberrant mRNA splicing has been shown to generate the production of a pathogenic HTT exon 1 fragment from the CAG-expanded *HTT* gene [68], which may evade RNAi effectors that act on mature mRNA transcripts. Depending on the timing of formation of this alternatively spliced mRNA variant, it may also avoid potential ASO binding. The CAG-expanded mRNA has itself been proposed as a source of toxicity in HD [69]; prevention of its formation would not be achieved by RNAi, although ASOs acting at the pre-mRNA level may be effective at reducing RNA-mediated toxicity. Both aberrant splicing and RNA toxicity could be eliminated, however, using genome editing approaches.

The extent to which these alternative pathways of toxicity exist and contribute to the disease phenotype in other forms of dementia could impact on the success of various gene suppression approaches for treatment.

### Assessment of target engagement

Interpretation of drug effect in early-stage, proof-of-concept clinical trials can be facilitated by measuring of the ability of the therapy to engage with its target. Methods for assessing target engagement include imaging modalities and measurement of target RNA or protein in an easily accessible biofluid or tissue sample. To assess target engagement directly, brain-imaging techniques that allow for quantitation of target protein in vivo are usually required. Recently, novel radiotracers for PET imaging have been developed to detect brain tau deposition [70], which may offer a direct assessment of target engagement by tau-lowering therapies. These techniques are relatively new, and identification of tracers with sufficient specificity to quantitate the protein of interest has proven challenging. As the field advances, PET imaging will probably become a very valuable tool for evaluation of target engagement of gene suppression therapies.

Quantification of target RNA or protein in a biofluid or tissue sample is another useful method for evaluating target engagement. For neurodegenerative conditions, where the target tissue is in the brain, collection of target tissue samples is impractical; however, collection of CSF samples is straightforward, and changes in neuronal proteins measured in CSF can be expected to reflect changes in brain tissue. Assays to detect CSF β-amyloid and tau protein are already used in the clinical evaluation of AD, and are incorporated into most clinical trials of AD. Likewise, assays to detect alpha-synuclein are sometimes used to assess target engagement in PD trials [71]. In the case of HD, an ultrasensitive single-molecule counting mHTT immunoassay has been developed that can accurately detect very low amounts of mHTT in the CSF. The level of mHTT in the CSF has been shown to correlate with disease stage and is also raised in pre-manifest HD [72].

It is worth noting that levels of target proteins in the CSF are an indirect reflection of absolute protein levels at actual sites of pathology in the brain parenchyma. However, using research in animals, it is possible to develop models of the relationship between drug pharmacokinetics (PK) and pharmacodynamics (PD) that predict target engagement in the brain regions of interest. Such models relate the reduction of expression of a gene in target tissue with parameters that can be measured in humans, such as the protein product of that gene’s expression in CSF. Studies in non-human primates, where brain anatomy is similar to humans, can provide a guide for interpretation of target engagement markers in biofluids. In fact, animal data were shown to translate well to humans during clinical development of nusinersen, an ASO for the treatment of patients with spinal muscular atrophy (SMA). In this programme, autopsy material was available from a few patients who received nusinersen during clinical development, and levels of ASO and target engagement markers in tissue were found to be well aligned with predictions from the pre-clinical PK–PD models, supporting the premise that thorough characterisation of drug pharmacology in relevant animal species is important for planning and interpretation of data in humans [73].

### Initiation of gene suppression therapies

As with any disease-modifying treatment for neurodegenerative disease, timing of intervention is crucial. In the case of HD, predictive genetic testing is available to those whose family history puts them at risk, and mutation carriers could potentially start gene suppression treatment before any clinical symptoms appeared. The goal would then be to initiate treatment just before the onset of pathology and prevent the disease from ever being manifest. Objective biomarkers are required to pinpoint pathology onset and when to start treatment. Large, prospective, longitudinal natural history and deep phenotyping studies have been undertaken in HD [74] and suggest that pathology may be detectable on volumetric neuroimaging 10.8 years prior to symptom onset [75–78]. Such observational studies are critical in other diseases to guide the timing of intervention with disease-modifying therapeutics to intercept early pathology.

## Allele specificity

ASOs can be developed to target mRNA from both the mutant allele and the wild-type allele equally or to favour the mutant allele over the wild-type allele. The former, non-allele-specific, approach has the advantages of addressing the entire HD population and maintaining flexibility for ASO design across the entire gene, increasing the likelihood of identifying a specific, potent and well-tolerated ASO. The potential disadvantage for non-allele-specific ASOs lies in reduction of the wild-type allele. An allele-specific approach can be achieved by targeting either the CAG expansion or a sequence containing a specific single nucleotide polymorphism (SNP) found only on the mutant allele. Allele-specific approaches have the advantage of preferential reduction of the mutant protein. Potential disadvantages include blunted potency and tolerability due to severe limitations on sequence space, undesirable reduction of other proteins containing CAG repeats not linked to disease (with CAG-targeting ASOs) and addressing only a subset of the disease population (with SNP-targeting ASOs).

In the case of HD, embryonic knockout of Htt is lethal [79] and its complete inactivation in the rodent brain causes a progressive neurological phenotype [80]. However, as mentioned previously, partial HTT lowering has been shown to be well tolerated in NHPs [30, 35] and complete inactivation of HTT in adulthood has been shown to be well tolerated in mice carrying the human *HTT* transgene [81]. Intuitively, strategies to silence the mutant allele whilst leaving the normal allele unaffected may be desirable to prevent any potential longer-term detrimental effects from loss of wild-type protein function. It is noteworthy, however, that ASOs do not ever achieve complete gene suppression. Even at high doses of ASO, residual HTT will be produced, mitigating risk of non-allele-specific approaches.

ASOs complementary to the expanded CAG have been produced which have up to 6-fold selectivity for mHTT over HTT [82–84]; selectivity is thought to arise as longer CAG transcripts provide binding sites for multiple ASOs. One potential drawback of this strategy is that other polyQ-containing proteins may be affected, and the potency of CAG targeting therapies is not as great as for other approaches. BioMarin Pharmaceuticals have, however, very recently carried out a study showing that an ASO with sequence (CUG)7 successfully targeted the expanded CAG repeat in mutant Htt mRNA in two different mouse models of HD and that this led to phenotypic improvement [85].

SNPs have been identified that reside only on the mutant HTT allele [86, 87], with siRNAs designed to target these specific SNPs. Three such SNPs have been identified that could be used to treat a majority of patients in Europe and the USA [88]. A recent study has shown the feasibility of allele selective suppression in HD patient primary ex-vivo myeloid cells when using siRNA to target one of these potential SNPs, although the other SNPs tested did not achieve any allele selectivity [89]. This was thought to be due to the nucleotide sequence and tertiary structure of the transcript surrounding the base mismatch, and therefore a lower proportion of HD patients have targetable SNPs using this siRNA approach than originally thought.

As mentioned previously, ASOs could also be used in a SNP-targeting approach. An advantage of ASOs over siRNAs is that they can target SNPs present in intronic sequences, due to their capacity to bind pre-mRNA in the nucleus rather than only mature mRNA transcripts in the cytoplasm. ASOs targeting mutant-allele linked SNPs have been designed that lead to 50-fold selectivity of the mHTT allele over the normal allele [90–92]. Three to five ASOs targeting different SNP sites would be needed to treat 80% of HD patients [93]. Wave Life Sciences are initiating clinical trials of two ASOs targeting SNP-containing regions of *HTT* mRNA; this involves screening potential trial subjects for the presence of these SNPs before allocation to one or other of the compounds [94].

## Recent and ongoing clinical trials of gene suppression

The first human clinical trial of intrathecal delivery of ASOs for the treatment of a neurodegenerative condition was carried out in patients with amyotrophic lateral sclerosis (ALS) [3]. Inherited mutations in SOD1 account for 13% of familial ALS and 2% of all ALS, and cause disease through a toxic gain-of-function of SOD1 protein. An ASO targeting SOD1 mRNA was shown to reduce both wild-type and mutant SOD1 (i.e. non-allele selective) in transgenic mice and in human cell models of ALS [95, 96]. A phase 1 trial carried out by Ionis Pharmaceuticals has since demonstrated the safety and tolerability of intrathecal infusion of this drug in ALS patients and has proven that the drug is distributed to spinal cord tissues following injection and is detectable in the CNS 3 months following injection [3]. Having demonstrated the feasibility of this approach, Ionis Pharmaceuticals, in conjunction with Biogen, is currently undertaking a phase 1/2a clinical study of a more potent ASO (ISIS-SOD1_Rx_) in ALS associated with a SOD1 mutation [97].

As mentioned previously, another phase 1/2a clinical trial is also currently being undertaken by Ionis to test the safety and tolerability of a MOE-modified ASO targeting human HTT delivered by lumbar intrathecal bolus administration to early-stage HD patients. The trial includes neuroimaging assessments and exploratory endpoints to assess the effect on cognitive, motor and neuropsychiatric symptoms [1]. A clinical trial of AAV2-delivered anti-HTT miRNA, which has shown promise in NHPs [29], is also being planned to initiate in the next few years [98].

Patients with SMA type 1 (infantile onset) have been treated with lumbar intrathecal bolus injections of an MOE-modified ASO (Nusinersen), and have shown dramatic improvement [34]. This is an important result for the field, but it is worth noting that Nusinersen differs mechanistically from other ASOs because it does not trigger RNAse H1-induced cleavage of the target mRNA, and therefore does not lead to “gene suppression”. Infants with SMA type 1 have homozygous gene mutations or deletion of *SMN1*, leading to a lack of survival motor neuron (SMN) protein and the death of motor neurons, which are particularly reliant on it. Nusinersen in fact alters the splicing of the mRNA of another gene, *SMN2*, that usually encodes a non-functional SMN protein, and in doing so increases the amount of functional SMN available in the cell. The phase 3 ENDEAR trial of this drug showed such promising efficacy in interim analysis that urgent work has already begun on an extended access programme to widen the availability of the drug to those not in a clinical trial [99]. The drug (marketed as Spinraza™) received expedited approval by the FDA [100] for all forms of SMA in paediatric and adult patients, and is administered by lumbar intrathecal bolus injection at 4-month intervals. The success of Nusinersen is encouraging; however, infantile SMA is an aggressive and rapidly progressive disease which manifests in the first few weeks or months of life, and is generally fatal over 1–2 years. Therefore positive treatment effects could be easily and rapidly discerned in the clinical trial setting. The outcome of gene suppression therapies for most other neurodegenerative diseases will most probably take longer to elucidate, as positive effects may not be so striking.

## Future gene suppression targets in neurodegeneration

Recently DeVos et al. [4] have shown that treatment with tau-reducing ASOs prevents neuronal loss in a mouse model of tauopathy. Tau is a microtubule-associated protein in neurons, which becomes hyper-phosphorylated, misfolds and forms insoluble neurofibrillary tangles (NFTs) in AD. The close temporal and spatial relationship between tau pathology and neurodegeneration is thought to underlie the widespread neuronal loss and progressive dementia seen in this condition. Indeed mutations in the *MAPT* gene, which encodes tau protein, are causative for another neurodegenerative disease—frontotemporal lobar degeneration (FTLD). This study demonstrated improved survival and histopathology in a mouse model of tauopathy, and also showed tau protein reduction in CNS tissues and CSF in non-human primates following intrathecal bolus administration of ASOs targeting *MAPT* mRNA. This opens up the exciting potential for gene suppression treatments of tau as a therapeutic avenue for AD and other tauopathies such as progressive supranuclear palsy (PSP), and clinical trials of tau-lowering therapies are imminent.

The possibility of treatment with ASOs that reduce pathogenic splice variants of *C9orf72* is also under investigation for the treatment of amyotrophic lateral sclerosis (ALS) and frontotemporal dementia (FTD). In the future, an ASO that suppresses alpha-synuclein gene (*SNCA*) expression to reduce alpha-synuclein protein might have utility in limiting the progression of PD [5]. In addition, an ASO targeting leucine-rich-repeat kinase 2 (*LRRK2*) (mutations of which are the major cause of familial late-onset PD) has been shown to reduce the spread of alpha-synuclein pathology in the mouse brain [6]. There may be great benefit in preventing the cortical dissemination of alpha-synuclein pathology, to target the cognitive decline and dementia that will eventually afflict more than 80% of PD patients and is one of the most feared consequences for those developing PD.

Spinocerebellar ataxia type 2 (SCA2) is an autosomal-dominant, adult-onset neurodegenerative disease caused by a polyglutamine expansion in the ataxin-2 gene (*ATXN2*). Scoles et al. [7] recently carried out a screen of multiple ASOs directed against *ATXN2* mRNA (non-allele specific) and tested their most promising candidate in two mouse models. Following intracerebroventricular injection, this ASO localised to Purkinje cells and reduced cerebellar *ATXN2* expression and levels of cerebellar ATXN2. This was also associated with significantly improved motor function [7].

An ASO lowering ATXN2 has also been shown to increase the lifespan and improve motor symptoms in a mouse model of TDP-43 proteinopathy [8]. Nearly all patients with ALS have aggregates of TDP-43 (an RNA-binding protein) in their brain and spinal cord, and TDP-43 pathology is also found in 50% of cases of FTD. Genetic lowering of ataxin-2 (using ataxin-2 knockout mice crossed with TDP-43 transgenic mice) leads to a reduction in TDP-43 aggregation. However, therapies that suppress TDP-43 are not viable due to the protein’s vital cellular functions. The finding that ATXN2 lowering markedly extends survival in these mice lends promise to this approach for the treatment of sporadic ALS and FTD, and is the first example of an ASO therapy targeting a modifier gene that is not directly causative of the disease [8].

## Conclusions

Gene suppression technologies have made enormous progress in the last 20 years. Clinical trials of ASOs are already underway and trials of RNAi and genome editing using ZFNs are not far behind. The relative advantages of ASOs and RNAi are summarised in Table 1. Lessons learnt from work carried out in the field of HD, as well as ALS and SMA, can be applied to other neurodegenerative diseases in which the causative gene mutation or disease protein is known.

**Table 1:** Relative advantages of RNAi and ASOs as a strategy to achieve gene suppression

Advantages of different approaches to post-transcriptional gene suppression	
RNA interference	Anti-sense oligonucleotides
• siRNAs do not cross the BBB and if introduced into CSF cannot achieve widespread distribution in the CNS parenchyma. However, siRNA effector sequences can be constitutively delivered through miRNA expression systems expressed from a viral vector. • siRNAs can lead to very high levels of translational suppression and lowering of target protein, if this is desired. • siRNAs have prolonged effects in terms of gene suppression and so there is potentially minimal or no need for repetitive administration. However, this is a type of gene therapy, which is irreversible, and there are no antidotes. • Through peripheral administration in neonates or very early infancy, global or larger CNS areas can be targeted.	• ASOs do not cross an intact BBB. However, ASOs are soluble in artificial CSF and can be delivered directly into the CSF space. Once introduced into the CSF, modified ASOs achieve widespread distribution in the CNS parenchyma and enter neuronal and glial cells. • ASOs have predictable, dose-dependent pharmacokinetics, enabling modulation of dose to achieve the desired level of pharmacological activity; with intrathecal bolus dosing every few months in symptomatic patients and potentially less frequently in pre-symptomatic patients. • ASO pharmacological effects are reversible and gene suppression reverses when treatment is stopped. • In addition to exonic regions, ASOs are able to target intronic regions as they bind to pre-mRNA rather than only mature transcripts. Thus they have more mRNA “real estate” from which to find the ideal ASO drug candidate, and can be used to treat a wider range of diseases. • Avoids saturation of RNAi pathways which can lead to liver toxicity. • No need for viral vector delivery, and therefore avoids the generation of an immune response.

*ASO* anti-sense oligonucleotide, *BBB* blood–brain barrier, *CSF* cerebrospinal fluid, *CNS* central nervous system, *miRNA* micro-RNA, *RNAi* RNA interference, *siRNA* small interfering RNA

Challenges with drug delivery of gene suppression agents have been researched extensively and largely overcome. In the case of ASOs, lumbar intrathecal bolus delivery is proving to be a viable option, and in the case of RNAi, modification of viral vectors has allowed for tolerability and efficacy. Administration of viral vectors either through directed brain injection/infusion to target specific sites, or through peripheral vascular injection leading to global CNS effects, has been tolerated in animal models.

The next few years will see further expansion in the clinical evaluation of gene suppression therapies for the treatment of dementia, both in terms of the number of neurodegenerative diseases targeted and the number of patients treated. Thus there is real hope that, in the face of aging populations and the ever-increasing incidence of dementia, we will in the near future be able to offer effective treatments to sufferers. Moreover, with advances in biomarker technology that can detect the increasingly understood evolution of pathology in asymptomatic individuals, the prospect of early disease interception to prevent the symptomatic expression of these neurodegenerative disorders is a tantalising possibility.

## Abbreviations

AAV: Adeno-associated virus
AD: Alzheimer’s disease
Ago2: Argonaut 2 protein
ALS: Amyotrophic lateral sclerosis
APP: Amyloid precursor protein
ASO: Anti-sense oligonucleotide
*ATXN2*: Ataxin-2
BBB: Blood–brain barrier
*C9Orf72*: Chromosome 9 open reading frame 72
CAG: Cytosine–adenine–guanine
CNS: Central nervous system
CRISPR: Clustered regularly interspaced short palindromic repeats
CSF: Cerebrospinal fluid
DARPP32: Dopamine and cAMP-regulated neuronal phosphoprotein
DNA: Deoxyribonucleic acid
FDA: Food and Drug Administration (US)
FTD: Frontotemporal dementia
FTLD: Frontotemporal lobar degeneration
gRNA: Guide RNA
HD: Huntington’s disease
HDV: Hepatitis delta virus
HEK293: Human embryonic kidney cells 293
HTT: Huntingtin protein
IFN: Interferon
IVI: Intravenous infusion
LRRK2: Leucine-rich-repeat kinase 2
LV: Lentivirus
MAPT: Microtubule associated protein tau
mHTT: Mutant Huntingtin protein
miRNA: Micro-RNA
mRNA: Messenger RNA
NFT: Neurofibrillary tangle
NHP: Non-human primate
PD: Parkinson’s disease
PKR: Protein kinase R
pol II/pol III: DNA polymerase II/polymerase III enzyme
PolyQ: Polyglutamine
ppENK: Preproenkephalin
pre-miRNA: Precursor micro-RNA
pri-miRNA: Primary micro-RNA
PrP: Prion protein
PSP: Progressive supranuclear palsy
RISC: RNA-induced silencing complex
RNA: Ribonucleic acid
RNAi: RNA interference
SCA2: Spinocerebellar ataxia type 2
shRNA: Small hairpin RNA
siRNA: Small interfering RNA
SMA: Spinal muscular atrophy
SNCA: Gene encoding alpha synuclein
SNP: Single-nucleotide polymorphism
SOD1: Superoxide dismutase 1
TALEN: Transcription activator-like effector nuclease
TLR: Toll-like receptor
ZFN: Zinc finger nuclease
ZFP: Zinc finger protein
ZFTR: Zinc finger transcriptional repressors
